# SARS-CoV-2 mRNA Dual Immunization Induces Innate Transcriptional Signatures, Establishes T-Cell Memory and Coordinates the Recall Response

**DOI:** 10.3390/vaccines11010103

**Published:** 2023-01-01

**Authors:** Ioanna Papadatou, Maria Geropeppa, Kleio-Maria Verrou, Marianna Tzanoudaki, Theano Lagousi, Emmanouil Liatsis, Vana Spoulou

**Affiliations:** 1 First Department of Paediatrics, Medical School, “Aghia Sophia” Children’s Hospital, National and Kapodistrian University of Athens, 11527 Athens, Greece; 2 Immunobiology and Vaccinology Research Lab, Medical School, National and Kapodistrian University of Athens, 11527 Athens, Greece; 3 University Research Institute of Maternal and Child Health and Precision Medicine, “Aghia Sophia” Children’s Hospital, National and Kapodistrian University of Athens, 11527 Athens, Greece; 4 Center of New Biotechnologies & Precision Medicine, Medical School, National and Kapodistrian University of Athens, 11527 Athens, Greece; 5 Department of Immunology and Histocompatibility, Specialized Center and Referral Center for Primary Immunodeficiencies, Paediatric Immunology, “Aghia Sophia” Children’s Hospital, 11527 Athens, Greece

**Keywords:** SARS-CoV-2 vaccine, immunological memory, transcriptomics, systems vaccinology

## Abstract

Background: mRNA vaccines have played a crucial role in controlling the SARS-CoV-2 global pandemic. However, the immunological mechanisms involved in the induction, magnitude and longevity of mRNA-vaccine-induced protective immunity are still unclear. Methods: In our study, we used whole-RNA sequencing along with detailed immunophenotyping of antigen-specific T cells and humoral RBD-specific response to dual immunization with the Pfizer–BioNTech mRNA vaccine (BNT162b2) and correlated them with response to an additional dose, administered 10 months later, in order to comprehensively profile the immune response of healthy volunteers to BNT162b2. Results: Primary dual immunization induced upregulation of the Type I interferon pathway and generated spike protein (S)-specific IFN-γ+ and TNF-α+ CD4 T cells, S-specific memory CD4 T cells, and RBD-specific antibodies against SARS-CoV-2. S-specific CD4 T cells induced by the primary series correlated with the RBD-specific antibody titers to a third dose. Conclusions: This study demonstrates the induction of both innate and adaptive immunity in response to the BNT162b2 mRNA vaccine in a coordinated manner and identifies the central role of primarily induced CD4+ T cells as a predictive biomarker of the magnitude of anamnestic immune response.

## 1. Introduction

Since December 2019, severe acute respiratory syndrome coronavirus 2 (SARS-CoV-2) has caused a global pandemic, resulting in more than 6 million deaths [1]. Vaccines against SARS-CoV-2 that elicit protective immune responses are crucial to the prevention of the morbidity and mortality caused by SARS-CoV-2 infection.

The development of highly effective vaccines is closely tied to the induction of robust and long-lived immunological memory. Although humoral response to SARS-CoV-2 mRNA vaccines has been extensively reported, studies on other components of the immune response to immunization have been scarce, and the mechanisms that determine the induction, the magnitude and the durability of the mRNA vaccine-induced immunity remain to be elucidated.

Moreover, mRNA vaccines against SARS-CoV-2 have demonstrated up to 95% efficacy in preventing severe COVID-19 [2]. Despite the proven efficacy, the conferred immunity is short-lived [3], and the identification of individuals who respond poorly to vaccination suggests a variability in the vaccine-induced immune response [4,5]. Therefore, the identification of biomarkers that can detect vaccinated individuals who will mount suboptimal responses is crucial for the optimization of tailored vaccination policies.

In this study, we utilized a systems vaccinology approach in order to comprehensively profile the BNT162b2-induced immune response, investigating both innate and adaptive immunity, and to identify early predictive markers for subsequent recall responses.

## 2. Materials and Methods

### 2.1. Study Population and Study Design

**Figure 1:**
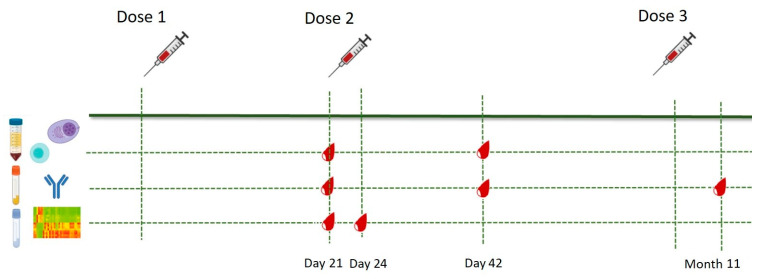
Immunization and sample collection schedule. Eligible participants received two primary doses of the mRNA BNT162b2 vaccine 3 weeks apart and an additional dose 10 months later. Blood samples for T cell phenotyping were collected prior to the second dose (Day 21) and three weeks after the second dose (Day 42). Sera for antibody analysis were collected at the abovementioned time-points, as well as three weeks after the anamnestic dose (Month 11). Blood samples for transcriptome analysis were obtained prior to and three days after the second dose (Day 21 and Day 24, respectively).

Eighteen SARS-CoV-2-naïve healthy healthcare professionals 28–65 years old were enrolled in the study between January and December 2021 under informed consent. Prior to enrollment, previous COVID-19 infection was excluded using an in-house-developed ELISA [6]. Subjects with major comorbidities (malignancies, immunosuppression, chronic kidney disease, liver failure, genetic syndromes) were excluded. All participants received two primary doses of the Pfizer–BioNTech mRNA BNT162b2 vaccine 3 weeks apart followed by a third dose 10 months later. Whole blood samples for peripheral blood mononuclear cell (PBMC) isolation were collected prior to the second dose (Day 21, D21) and three weeks after the second dose (Day 42, D42); samples for transcriptome analysis were collected on D21 and three days after the second dose (Day 24, D24); sera for RBD-specific antibody enumeration were collected on Days 21 and 42 and 3 weeks after the third dose (Month 11). The immunization schedule and sample collection time-points are illustrated in Figure 1.

### 2.2. Sample Collection and Storage

Sera isolated from venous blood were stored at −20 °C. PBMCs were isolated from 20 mL of heparinized whole blood via density gradient centrifugation and stored at −80 °C. PBMCs were thawed with RPMI 1640 at 37 °C and allowed to rest for 12–16 h in cell resting buffer (90% IMDM/10% FBS) at 37 °C and 5% CO_2_. Total RNA was isolated from whole blood, using the Tempus™ Spin RNA Isolation Kit (Thermofisher, Waltham, MA, USA).

### 2.3. Whole RNA Sequencing and Differential Expression Gene (DEG) Analysis

Purification of mRNA from total RNA was carried out with the PAXgene Blood RNA kit (Qiagen, Hilden, Germany). After fragmentation and priming of mRNA, quantification was applied using the NanoDrop ND-1000 (Thermofisher, Waltham, MA, USA) and Bioanalyzer RNA 6000 Nano assay (Agilent). A total of 500 ng of RNA per sample of sufficient quality was processed using the QuantSeq 3′ mRNA-Seq Library Prep Kit FWD (Lexogen, Wien, Austria) for library preparation. The libraries were assessed for molarity and median library size using Bioanalyzer High Sensitivity DNA Analysis. After multiplexing and addition of 5% PhiX Control v3 (Illumina, San Diego, CA, USA), as spike in, NGS was performed on a NextSeq550 Platform (Illumina), with a NextSeq 500/550 High Output Kit v2.5, 150 Cycles, single read. Overall, >335 million reads were generated.

The quality of the FASTQ files was assessed using FastQC (version 0.11.9; https://www.bioinformatics.babraham.ac.uk/projects/fastqc; accessed on 10 November 2021). The reads were mapped to the GRCh38 reference human genome using STAR [7]. After quality control, raw bam files were summarized to a 3′UTR read counts table, using the Bioconductor package GenomicRanges [8], through metaseqR2 [9]. Bioconductor package DESeq2 was used to normalize gene counts and perform DEG analysis [10]. The adjusted *p*-value (p.adj) was used as the statistical significance metric for DEGs. A volcano plot was constructed through the ggplot2 R package (v.1.0.12). RNA sequencing data have been deposited in the Gene Expression Omnibus Database (record GSE205402).

### 2.4. Ex Vivo Spike (S)-Protein Stimulation of PBMCs and Flow Cytometry Analysis

10^6^ thawed PBMCs were seeded in a 96-well plate in a total volume of 100 μL cell culture medium. Next, cells were stimulated for 2 h with SARS-CoV-2 S-protein PepTivator^®^ SARS-CoV-2 Prot_S—research grade 6 nmol/peptide (#130-126-700) (Miltenyi, Bergisch Gladbach, Germany), containing a pool of lyophilized peptides, consisting mainly of 15-mer sequences with 11 amino acids (aa) overlap, covering the immunodominant sequence domains of the surface glycoprotein (“S”) of SARS-CoV-2. DMSO and Cytostim™ (Miltenyi) were used as negative and positive controls respectively. After the addition of Brefeldin A, cells were further incubated for 4 h and then stained with the Viobility™ 405/452 Fixable Dye, fixed, and permeabilized for intracellular staining. Cells were stained with fluorophore-conjugated antibodies against CD3, CD4, CD8, IFN-γ, TNF-α, CD14, CD20 and CD154 (included in the SARS-CoV-2 Prot_S T Cell Analysis Kit (PBMC) human, #130-127-586) (Miltenyi), alongside CCR7 and CD45RA (BioLegend, San Diego, CA, USA). Data were acquired on an 8-laser Navios Flow Cytometer and analyzed using Kaluza 2.1.1, after subtraction of negative control. The gating strategy is detailed in Supplementary Figure S1.

### 2.5. Enzyme-Linked Immunosorbent Assay (ELISA)

ELISA 96-well plates were coated with recombinant SARS-CoV-2 RBD diluted in PBS and incubated for 2 h at room temperature (RT). After blocking with PBS-Tween and 3% BSA for 1 h at RT, sera were added and incubated for 2 h at RT. Goat anti-human alkaline phosphatase-conjugated IgG antibody (Jackson ImmunoResearch Laboratories, West Grove, PA, USA) was added (1:3000) and antibody binding was detected using the substrate 4-nitrophenyl-phosphate-disodium salt hexahydrate (Sigma Chemicals, Saint Louis, MO, USA) at 405 nm. Results are reported in U/mL of IgG [6].

### 2.6. Statistical Analysis

Continuous variables are presented as mean or median values. All comparative statistical analyses were performed using either a two-sided *t* test when the variable was normally distributed, or a nonparametric test if not. Relationships were assessed using mono- or multi-variant analysis methods. Statistical significance was set at *p* = 0.05 and analyses were conducted using GraphPad (v6).

## 3. Results

During the study period, 18 SARS-CoV-2-naïve healthcare professionals were recruited for the purposes of the study. The participants’ demographic characteristics are described in Table 1.

### 3.1. Transcriptional Signature of the Immune Response to BNT162b2

**Figure 2:**
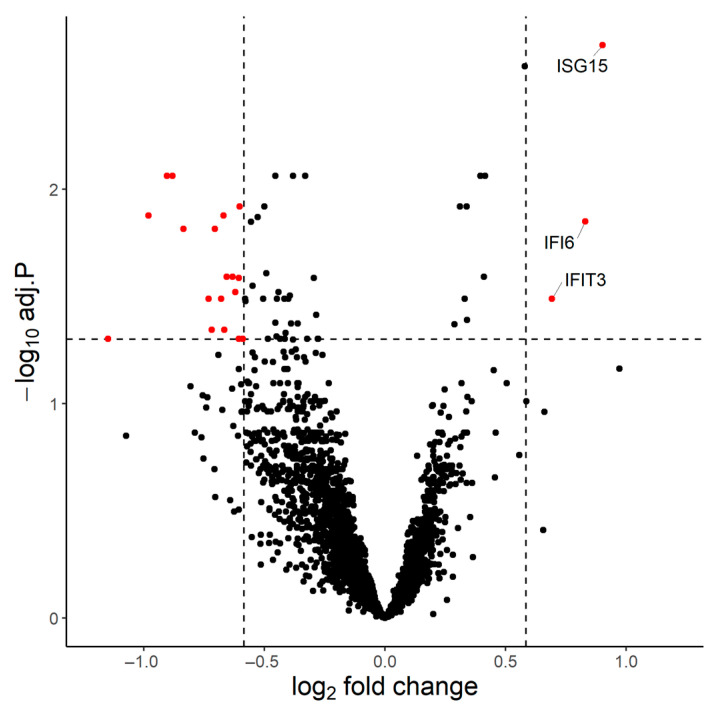
Innate antiviral immune response induced by dual mRNA BNT162b2 immunization. Volcano plot of the differentially expressed genes on D24 compared to D21 (p.adj < 0.05 and Fold Change > 1.5 for the upregulated genes, p.adj < 0.05 and FC < 1.5 for the downregulated genes). Three genes were upregulated (IFI6, IFIT3, ISG15), all involved in antiviral immunity and type I interferon pathways.

We first performed bulk mRNA sequencing of whole blood samples from 15 individuals collected on Days 21 and 24. Four out of thirty samples did not pass quality control and were removed from the analysis. On D24 in comparison to D21, 3 genes (IFI6, IFIT3, ISG15) were found upregulated (p.adj < 0.05 and fold change (FC) > 1.5) and 18 (AC104389.5, ALPL, BASP1, CHI3L1, CSF2RB, CXCR2, GCA, KCNJ15, MGAM, MME, NAMPT, NIBAN1, NKX3-1, RGS2, SEC14L1, ST20-MTHFS, SVBP, THBD) downregulated (p.adj < 0.05 and FC < 1.5) (Figure 2).

Interferon alpha-inducible protein 6 (IFI6), interferon-induced protein with tetratricopeptide repeats 3 (IFIT3) and ubiquitin cross-reactive protein (ISG15) have strong interactions within the biological pathways ‘Type I interferon signaling pathway’ (GO: 0060337) and ‘Defense response to virus’ (GO: 0051607), highlighting the involvement of the innate system in the immune response to BNT162b2.

STRING analysis showed that the 18 downregulated genes are biologically unlinked; thus, their upregulation has minimum biological significance.

### 3.2. T Cell Response to BNT162b2

We then investigated the S-specific T cell response to primary dual immunization with BNT162b2 by a flow cytometric analysis using PBMCs stimulated in vitro with the SARS-CoV-2 spike peptide pool (PepTivator^®^, Miltenyi) in 18 participants on Days 21 and 42.

**Figure 3:**
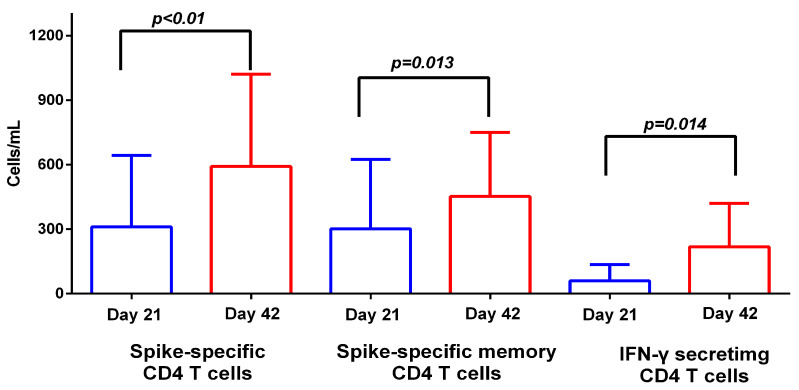
Cellular response to BNT162b2. Spike-specific CD4 T cells, spike-specific CD4 memory T cells and IFN-γ-secreting CD4 T cells before (D21) and 21 days (D42) after the second BNT162b2 dose in SARS-CoV-2 naïve individuals. Data are represented as mean values with standard deviation.

S-specific CD4 T cells (CD3 + CD4 + CD154+) and S-specific CD4 memory T cells (MTCs) (CD3 + CD4 + CD154 + CD45RA−) increased significantly on D42 (mean values 310.0 vs. 591.7 cells/mL, *p* < 0.01 and 301.0 vs. 452.0 cells/mL, *p* = 0.013, respectively) (Figure 3). Of note, background upregulation levels were low, suggesting that CD4 T cells that were CD154+ were indeed S-specific.

IFN-γ (CD3 + CD4 + IFN-γ+)-secreting CD4 T cells were detectable on D21 and significantly increased on D42 (mean values 60.0 vs. 218.0 cells/mL, *p* = 0.014) (Figure 3). Similarly, TNF-α (CD3 + CD4 + TNF-α+)-secreting CD4 T cells were detectable on D21 but did not increase significantly on D42 (median values 70.8 vs. 124.2 cells/mL, *p* = 0.1). Immunization with the second dose of BNT162b2 induced the production of IFN-γ- and TNF-α-secreting cells in a coordinated manner, with the two T cell subsets positively correlated on D42 (r = 0.92, *p* < 0.001; Supplementary Figure S2). Polyfunctional CD4 T cells (CD3 + CD4 + IFN-γ + TNF-α+) were detectable on D21 and increased on D42, though without statistical significance (median values 49.62 vs. 86.31 cells/mL, *p* = 0.1).

In contrast to the rapid and universal induction of S-specific CD4 T cells, SARS-CoV-2-specific CD8 T cell responses developed in low frequencies and with greater interpersonal variability. Only 30% of subjects generated detectable S-specific CD8 T cell responses following the first dose. These CD8 T cell responses were boosted by the second dose with 45% of subjects having detectable CD8 counts with a variable magnitude of response (5.66 to 107.89 cells/mL) (Supplementary Figure S3).

### 3.3. Antibody Response to BNT162b2

**Figure 4:**
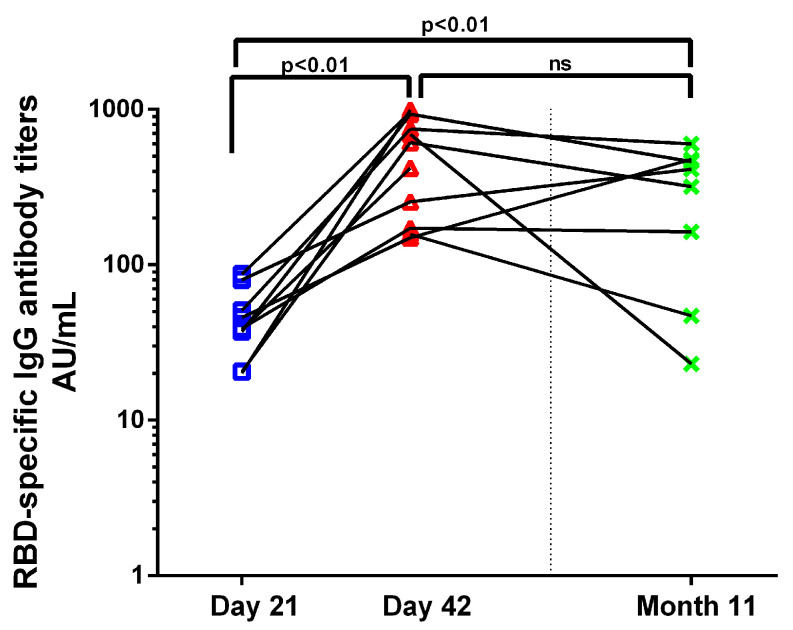
Humoral response to BNT162b2. Kinetics of RBD-specific IgG antibody titers before (D21) and 3 weeks (D42) after the second BNT162b2 dose and 3 weeks after an anamnestic dose (Month 11) in vaccinated individuals. Each dot represents a serum sample.

RBD-specific IgG antibodies (RBD-IgG) were measured by ELISA on Days 21, 42 and three weeks post a third BNT162b2 dose given 10 months after the first dose (Month 11), when it first became available for health-care professionals in Greece. RBD-IgG increased significantly on D42 and in Month 11 compared to D21 (38.06 AU/mL vs. 554.3 AU/mL, *p* < 0.01; 38.06 AU/mL vs. 312.9 AU/mL, *p* < 0.01) (Figure 4). RBD-IgG achieved after the recall dose were equivalent to the titers post the primary series.

### 3.4. Correlation between Cellular and Humoral Response to BNT162b2

We next sought to identify potential biomarkers that could predict the magnitude of immune response to primary and recall vaccination. With regards to antibody titers, RBD-IgG on D21 were identified as the best predictive biomarker in our cohort, as RBD-IgG on D21 were positively correlated with total S-specific CD4 T cells on D21 (r = 0.62, *p* = 0.04) and on D42 (r = 0.8, *p* < 0.01), as well as with S-specific CD4 MTCs on D21 (r = 0.64, *p* = 0.03) and on D42 (r = 0.66, *p* = 0.02) (Supplementary Figure S4). In addition, antibody titers on D21 were positively correlated with RBD-IgG in Month 11 (r = 0.74, *p* = 0.035; Supplementary Figure S3). In contrast, RBD–IgG on D42 demonstrated less interpersonal variability (GMT 360.9 AU/mL, 95% CI 215.5–604.4) than on D21 (GMT 17.8 AU/mL, 95% CI 4.6–69) and no correlation was observed with either total T cell or MTC response.

**Figure 5:**
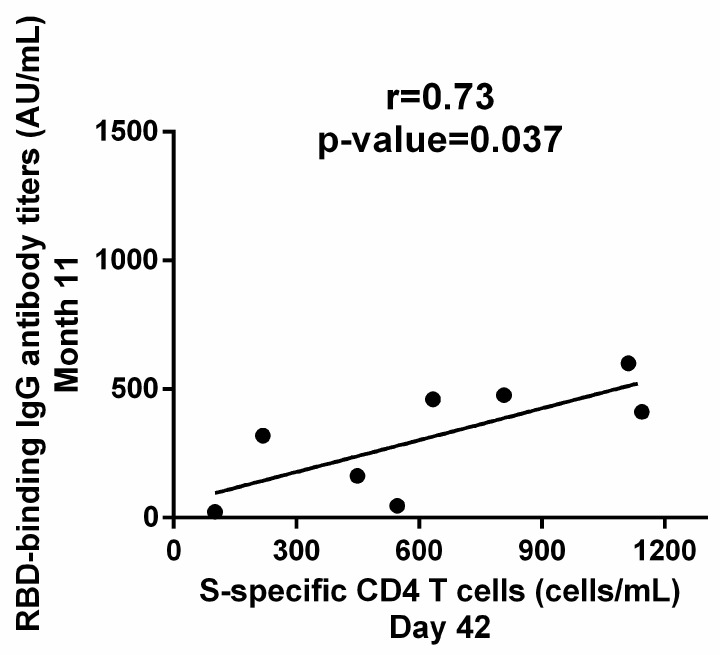
Correlation of cellular immunity and humoral immunity to BNT162b2. Cellular immunity after the completion of the primary dual immunization is correlated with the recall response to anamnestic vaccine dose, as S-specific CD4 T cells on D42 were correlated with RBD-binding IgG antibody titers on Month 11. Correlation was estimated with the Pearson r correlation coefficient and significance was calculated by a two-tailed *p* value. Each dot represents a serum sample.

Subsequently, we investigated whether cellular response to the primary series could also predict the magnitude of the immune response to the third dose. Indeed, S-specific CD4 T cells on D42 were positively correlated with RBD-IgG on Month 11 (r = 0.73, *p* = 0.037) (Figure 5). S-specific CD4 T cells on D21 did not correlate with humoral immunity at any time-point. Therefore, our results suggest that the optimal potential predictive biomarkers for the response to subsequent exposure to SARS-CoV-2 antigens in our cohort are RBD-IgG on D21 and S-specific CD4 T cells on D42.

**Figure 6:**
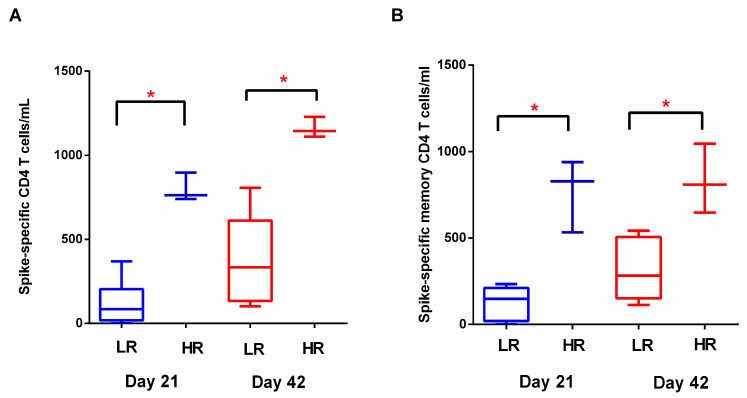
Humoral response after the first BNT162b2 dose may reflect the magnitude of cellular immunity. High responders (HR) (RBD-IgG > 50 AU/mL on D21) developed higher frequencies of (**A**) spike-specific CD4 T cells and (**B**) spike-specific memory CD4 T cells on either D21 or D42 compared to low responders. (LR). Data are represented as median values (min to max). *: *p*-value < 0.05.

Although mRNA vaccines against SARS-CoV-2 are considered highly effective for the prevention of severe COVID-19, current evidence suggests a heterogeneity of the immune response to BNT162b2 immunization, as studies have identified vaccinated individuals who do not mount efficient immune responses [5]. We postulated that humoral and cellular immunity to vaccination are induced in a coordinated manner. Therefore, we investigated whether “low responders” and “high responders” based on antibody titers on D21 also displayed distinct cellular and anamnestic immune responses. For that purpose, we set a cut-off point at the lower 25% of the RBD-IgG range in our cohort and stratified subjects to either the high- or low-responder group. After stratification according to humoral response following the first BNT162b2 dose, three subjects were stratified into high responders and eight subjects were stratified into low responders. Total S-specific and S-specific memory CD4 T cells differed significantly on D21 (median values 84.5 vs. 762.3 cells/mL, *p* < 0.05; 147 vs. 827 cells/mL, *p* < 0.05 respectively) and D42 (median values 333.3 vs. 1144 cells/mL, *p* < 0.05; 308.8 vs. 834 cells/mL, *p* < 0.05, respectively) (Figure 6). Therefore, we observed a vaccine-induced coordinated immune response, as vaccinated individuals who achieved higher antibody titers after the first vaccine dose also displayed a more robust cellular response. RBD-IgG titers following the third dose also differed between ‘low’ and ‘high’ responders (GMT 193.3 vs. 450.4 AU/mL), although these differences did not reach statistical significance.

## 4. Discussion

In this study, we performed an in-depth investigation of the immune response to the BNT162b2 vaccine in order to elucidate the mechanisms behind the development of innate and adaptive immune response to mRNA vaccines, involving transcriptional profiling, T cell phenotypic analysis and antibody kinetics pre- and post-vaccination. Our data collectively imply that a dual primary BNT162b2 series induces both innate and adaptive immune responses in a coordinated manner and identifies CD4+ T cells and antibody titers at specific time-points as predictive biomarkers of the magnitude of anamnestic immune response.

With regards to innate immunity, bulk transcriptomic analysis showed that BNT162b2 immunization stimulated targeted antiviral immunity with exclusive upregulation of the Type I interferon (IFN-I) pathway. More specifically, only three genes were significantly upregulated in response to the second dose in the present study, all involved in antiviral immunity and IFN-I pathways. IFIT3 is involved in the innate immune response to viral infection, as it regulates the fusion of the virus to endocytic vesicles and inhibits virus membrane fusion, in order to prevent the release of viral particles into the cytoplasm and control viral spread [11]. IFI6 is an IFN-stimulated gene (ISG) whose expression is highly regulated by the stimulation of IFN-I-alpha, which restricts various kinds of virus infections by targeting different stages of the viral life cycle [12], while ISG15 is upregulated in response to type I and type III IFN and leads to the conjugation of lysine residues of target proteins, a process termed ISGylation, with widely recognized antiviral activity [13]. Exogenous mRNA, such as the BNT162b2 vaccine, activates various endosomal and cytosolic innate sensors that form a critical part of the innate immune response to viruses leading to the production of IFN-I and multiple other inflammatory mediators [14]. Our findings are in accordance with existing knowledge, as recent studies also demonstrated significant enrichment of the IFN-I pathway and innate antiviral activity following secondary BNT162b2 immunization [15,16]. The 18 downregulated genes were found to be biologically unlinked, implying that their differential expression has little, if any, biological significance, as these genes are mostly involved in erythrocyte functions and metabolic pathways, such as activity of alkaline phosphatase, endoplasmic reticulum stress response and endothelial cell and neutrophil migration [17,18,19,20,21]. In contrast to Arunachalam et al., we were unable to identify any correlation between the innate transcriptional signatures and adaptive immune response or age, most probably due to the small size of our cohort [15]. Overall, our transcriptomic immune profiling of the immune response to BNT162b2 contributes to the limited data on the innate immune response to mRNA vaccines.

Subsequently, we investigated the adaptive immune response to BNT162b2 immunization. Our results demonstrate that both humoral and cellular responses were induced after two doses of the BNT162b2 vaccine and, more specifically, antibody titers as well as total S-specific and memory S-specific CD4 T cells significantly increased following the second vaccine dose. Achieving high titers of circulating antibodies is of great significance for the prevention of infection [22]. However, vaccine-induced humoral immunity has been proven to rapidly wane in the following months after immunization [23]. On the other hand, the generation of robust cellular immunity is thought to be an important factor for the longevity of vaccine-conferred protection, and MTCs induced by vaccination have proven to be exceptionally durable for other vaccines that confer life-long immunity in real-life settings, such as yellow fever and smallpox [24,25]. In regards to SARS-CoV-2 mRNA vaccines, previous studies have shown that MTC responses are sustained long after antibodies decline to baseline levels [26]. This durable cellular immunological memory may be responsible for continued protection against severe disease in vaccinated individuals, despite the rapid decline of antibody titers, and may explain the differences in vaccine efficacy against severe disease assumed to be mediated by MTCs [22,27]. Moreover, it has been shown that cellular response is significantly more resilient to SARS-CoV-2 variants of concern (VOCs) than neutralizing antibodies, and therefore vaccine-induced cellular immunity may offer protection across different waves of the pandemic [26,28].

Furthermore, we identified potential predictive biomarkers for the amplitude of recall responses. In our cohort, CD4 T cells induced by the primary series correlated with the humoral response to a third dose, highlighting the role of T cells in the development of antigen-specific immunological memory and the induction of recall responses upon re-exposure to SARS-CoV-2 antigens. In this context, antigen-specific T cell responses may be a more accurate correlate of vaccine-induced durable protection against severe disease and death, as has been previously shown for immunity conferred by natural SARS-CoV-2 infection [29,30,31]. Our findings are also in accordance with recent reports, where pre-existing cellular immunity, either by natural infection or immunization, is correlated with recall responses, as Goel et al. reported that Tfh cells induced by a first BNT162b2 dose correlated with antibodies achieved up to 6 months after the second dose [26], while Casado et al. reported that pre-existing CD4 deriving from previous infection or cross-reaction with other coronaviruses correlated positively with specific IgG-antibody titers after the first and second vaccine doses [32]. These findings highlight the ability of the immune system to mount primary and recall responses after exposure to SARS-CoV-2 antigens in a similar manner.

Altogether, our data add to the accumulating evidence of the significance of cellular response in vaccine-induced immunity against SARS-CoV-2. However, establishing antigen-specific T cells as a correlate of protection for SARS-CoV-2 vaccines may prove unrealistic in a large-scale real-life setting, as the assays are costly and laborious. In this study, we found that RBD-IgG induced by the first BNT162b2 dose was predictive of cell-mediated and antibody response to subsequent vaccine doses, and, therefore, antibody titers achieved after the first vaccine dose may be utilized as an easily reproducible predictive biomarker. We also used RBD-IgG titers after the first dose to identify high and low responders to vaccination and investigated whether cellular response was differentially induced. Indeed, low responders based on antibody titers also displayed lower frequencies of total S-specific and memory S-specific CD4 T cells, implying a coordination of cellular and humoral immune response to vaccination. Detection of low responders to vaccination with an easily measured biomarker has significant implications for vaccination policies, as these individuals may benefit the most from additional doses. Although repeated booster immunizations have been implemented in immunization schedules as a countermeasure to waning of vaccine-induced humoral immunity and high rates of SARS-CoV-2 infection in vaccinated individuals, this may not be a sustainable policy longitudinally. Taking into consideration the reported variability in the duration of vaccine-induced immune responses [33], heterologous vaccination may serve as an alternative strategy for the extension of the magnitude and longevity of vaccine-induced protection, as recent studies suggest that heterologous immunization may foster enhanced immunological responses [34,35].

The following study limitations need to be mentioned. Our study included a small number of participants, yet a substantial number for a high-depth immune profiling study. T cell markers CD45RA and CCR7 were included in the flow cytometry panel for the characterization of central and effector MTCs. However, results about CCR7 expression could not be extracted due to suboptimal flow cytometry images. Therefore, memory cells were solely gated based on the expression of CD45RA. Additionally, our cohort is skewed towards young, healthy adults; as such, our results may not fully represent the durability of vaccine-induced immunity in high-risk individuals. Further studies are required to clarify the immunological mechanisms involved in long-lasting immunity in these populations.

## 5. Conclusions

Collectively, our study describes the ability of the BNT162b2 vaccine to elicit a coordinated innate and adaptive response in a cohort of healthy adults. Of note, BNT162b2 stimulated genes involved in antiviral immunity and IFN pathways. The primary-recall immunization schedule induced humoral and cellular responses in a coordinated manner, while S-specific CD4 T cells after the completion of the primary series correlated with antibody titers achieved after the anamnestic dose. RBD-specific IgG antibodies after the first BNT162b2 dose could serve as a predictive marker for the development of robust immunity to subsequent doses. The unravelling of the immune response to mRNA vaccines may eventually facilitate the optimization of personalized vaccination strategies against SARS-CoV-2.

## Figures and Tables

**Table 1:** The demographic characteristics of the enrolled participants.

*N*	18
*Age*	
Mean (in years)	41.46
SD (in years)	12.36
*Gender*	
Female (%)	13 (72.2%)
Male (%)	5 (27.8%)
*Body Mass Index (BMI)*	
Mean (SD)	25.3 (4.0)

## Data Availability

Further information about data supporting the reported results will be provided by the corresponding author upon reasonable request.

## Supplementary Materials

The following supporting information can be downloaded at: https://www.mdpi.com/article/10.3390/vaccines11010103/s1, Figure S1: Gating strategy of spike-specific CD4+ T cells; Figure S2: Coordinated induction of IFN- and TNF-α producing CD4+ T cells after completion of the primary series; Figure S3: Spike-specific CD8 T cell response following dual BNT162b2 immunization; Figure S4: RBD-specific IgG antibody titers achieved after the first dose of the mRNA BNT162b2 vaccine may serve as a predictive marker of immune response to subsequent vaccine doses; Table S1: Monoclonal antibody fluorochromes included in the flow cytometry panel.
