# Supplementary material for: SARS-CoV-2 mRNA Dual Immunization Induces Innate Transcriptional Signatures, Establishes T-Cell Memory and Coordinates the Recall Response
Source: Vaccines (Basel). 2023 Jan 1;11(1):103. doi: 10.3390/vaccines11010103 (PMC9861479; doi:10.3390/vaccines11010103)
Supplement: Supplementary file 1 [file vaccines-11-00103-s001.zip › vaccines-2119891-supplementary.pdf]

SUPPLEMENTARY MATERIALS

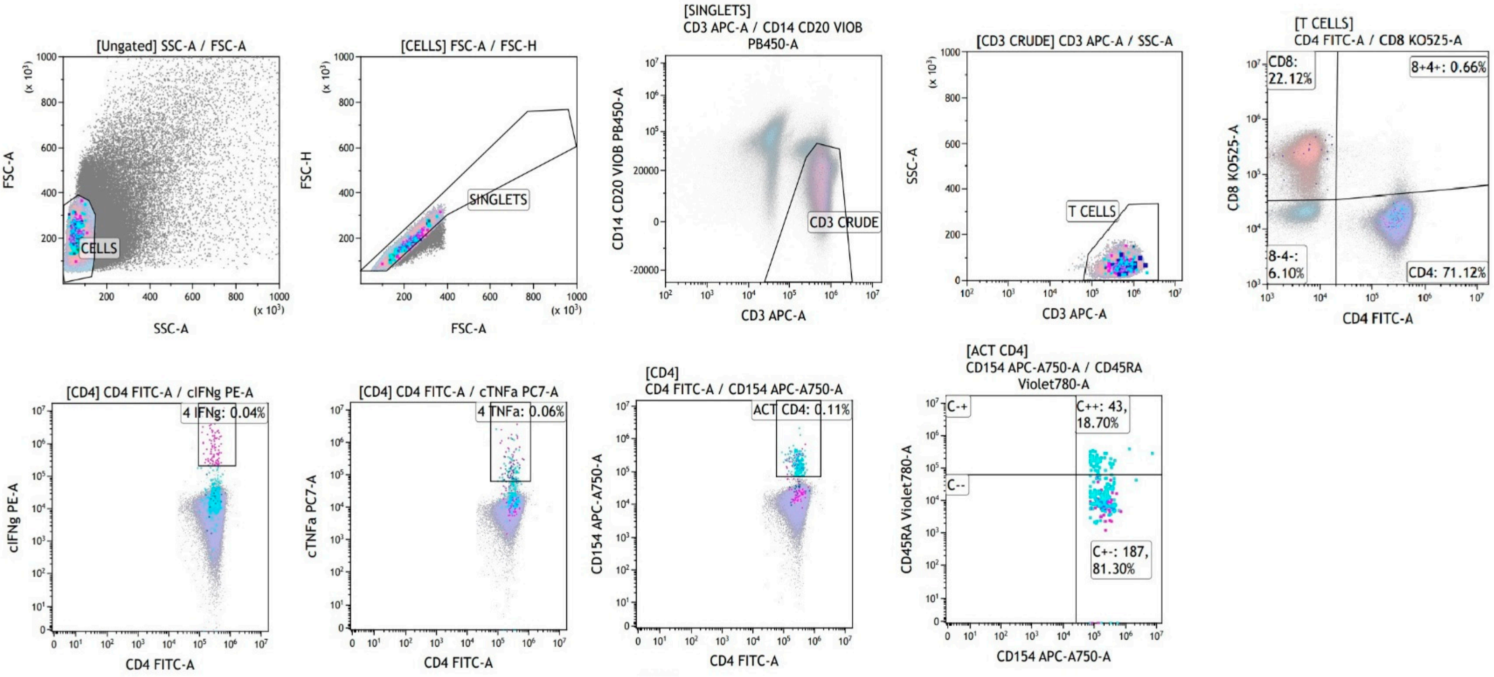

Supplementary Figure S1. Gating strategy of spike-specific CD4+ T cells

Flow cytometric characterization of SARS-CoV-2 spike-specific CD4 cells in peripheral blood mononuclear cells (PBMCs) isolated from SARS-CoV-2 naïve healthcare professionals immediately before and 21 days after the second dose of the mRNA BNT162b2 vaccine.

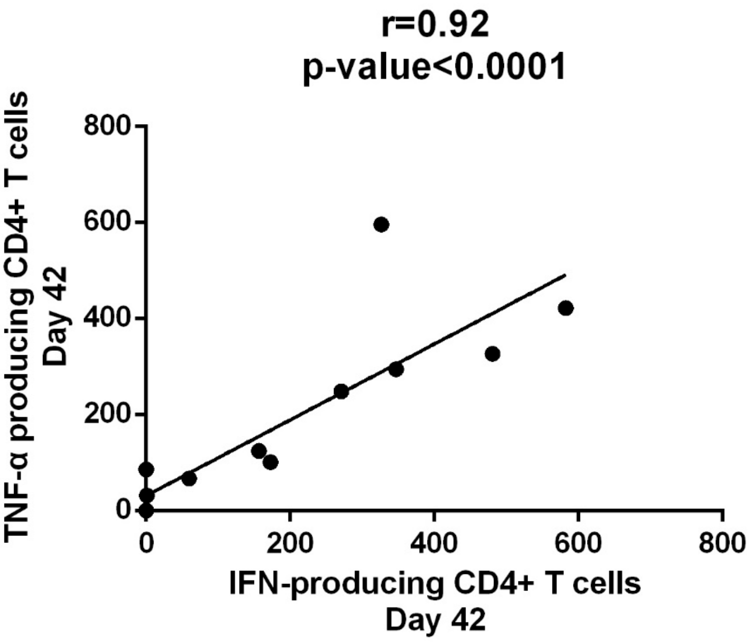

Supplementary Figure S2. Coordinated induction of IFN- and TNF-α producing CD4+ T cells after completion of the primary series.

IFN-producing CD4 T cells on Day 42 were correlated with TNF-α producing CD4 T cells at the same time-point. Correlation was estimated with Pearson r correlation coefficient and significance was calculated by a two tailed p value. Each dot represents a serum sample.

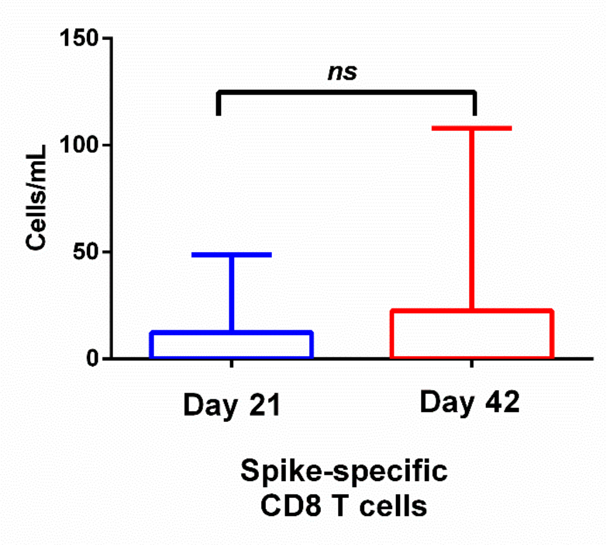

Supplementary Figure S3. Spike-specific CD8 T cell response following dual BNT162b2 immunization

Spike-specific CD8 T cells before (D21) and 21 days (D42) after the second BNT162b2 dose in SARS-CoV-2 naïve individuals.
Data are represented as minimum to maximum values.

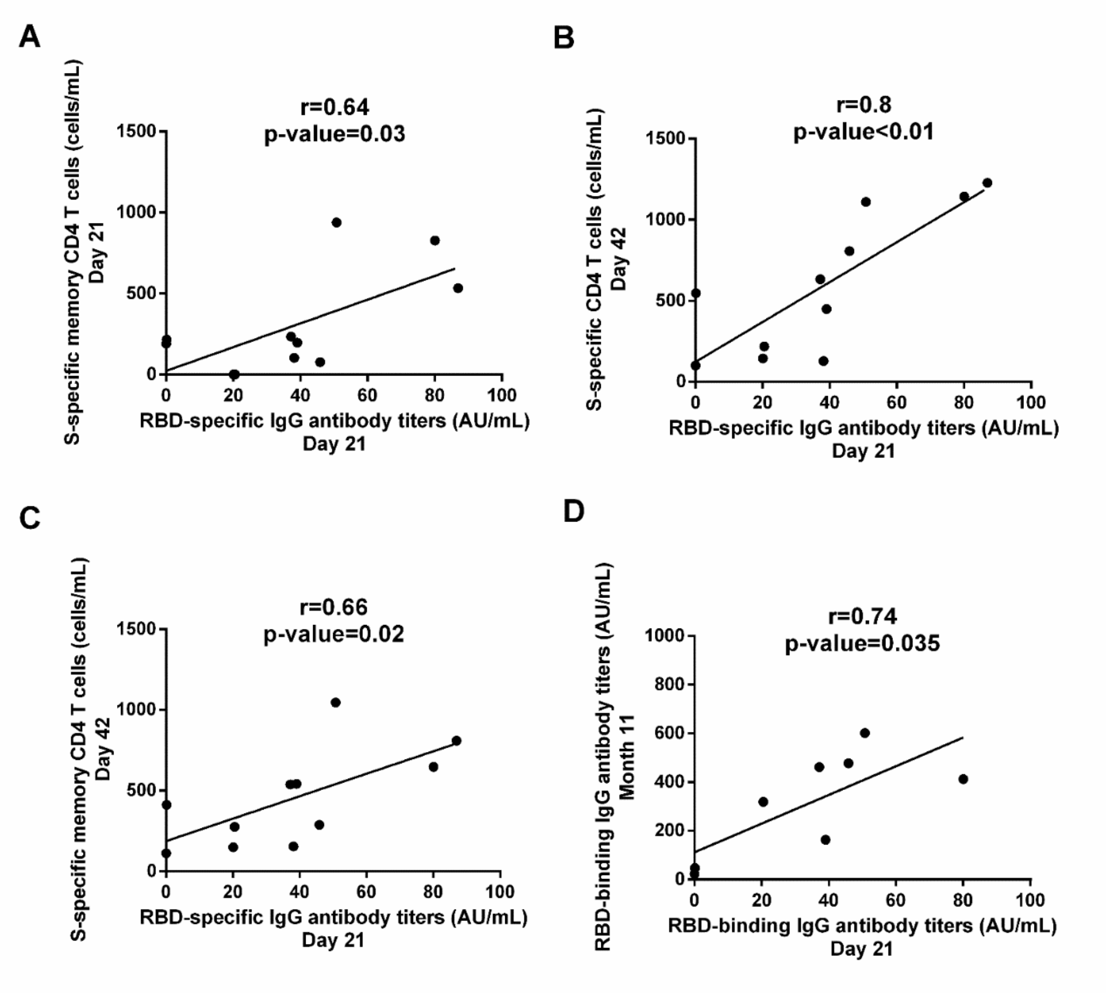

Supplementary Figure S4. RBD-specific IgG antibody titers achieved after the first dose of the mRNA BNT162b2 vaccine may serve as a predictive marker of immune response to subsequent vaccine doses.

Correlations of RBD-specific IgG antibody titers after the first dose of the vaccine (Day 21) with A) S-specific memory CD4 T cells on Day 21, B) S-specific CD4 T cells after the second dose (Day 42), C) S-specific memory CD4 T cells on Day 42 and D) RBD-specific IgG antibody titers after the third dose (Month 11). Correlations were estimated with Pearson r correlation coefficient and significance was calculated by a two tailed p value. Each dot represents a serum sample.

Antibodies	Supplier
APC anti-human CD3 REAfinity™ (clone REA613)	Miltenyi
Vio® Bright B515 anti-human CD4 REAfinity™ (clone REA623)	Miltenyi
VioGreen™ anti-human CD8 REAfinity™ (clone REA734)	Miltenyi
PE anti-human IFN-γ (clone 45-15)	Miltenyi
PE-Vio®-770 anti-human TNF-α (clone cA2)	Miltenyi
VioBlue® anti-human CD14 (clone TÜK4)	Miltenyi
VioBlue® anti-human CD20 (clone LT20)	Miltenyi
APC-Vio®-770 anti-human CD154 (clone 5C8)	Miltenyi
Brilliant Violet 650 anti-human CD197 (CCR7, clone G043H7)	BioLegend
Brilliant Violet 785 anti-human CD45RA (clone HI100)	BioLegend

Supplementary Table S1. Monoclonal antibody fluorochromes included in the flow cytometry panel
